# Supplementary figures and images for: Genetic effect on free amino acid contents of egg yolk and albumen using five different chicken genotypes under floor rearing system
Source: PLoS One. 2021 Oct 8;16(10):e0258506. doi: 10.1371/journal.pone.0258506 (PMC8500412; doi:10.1371/journal.pone.0258506)

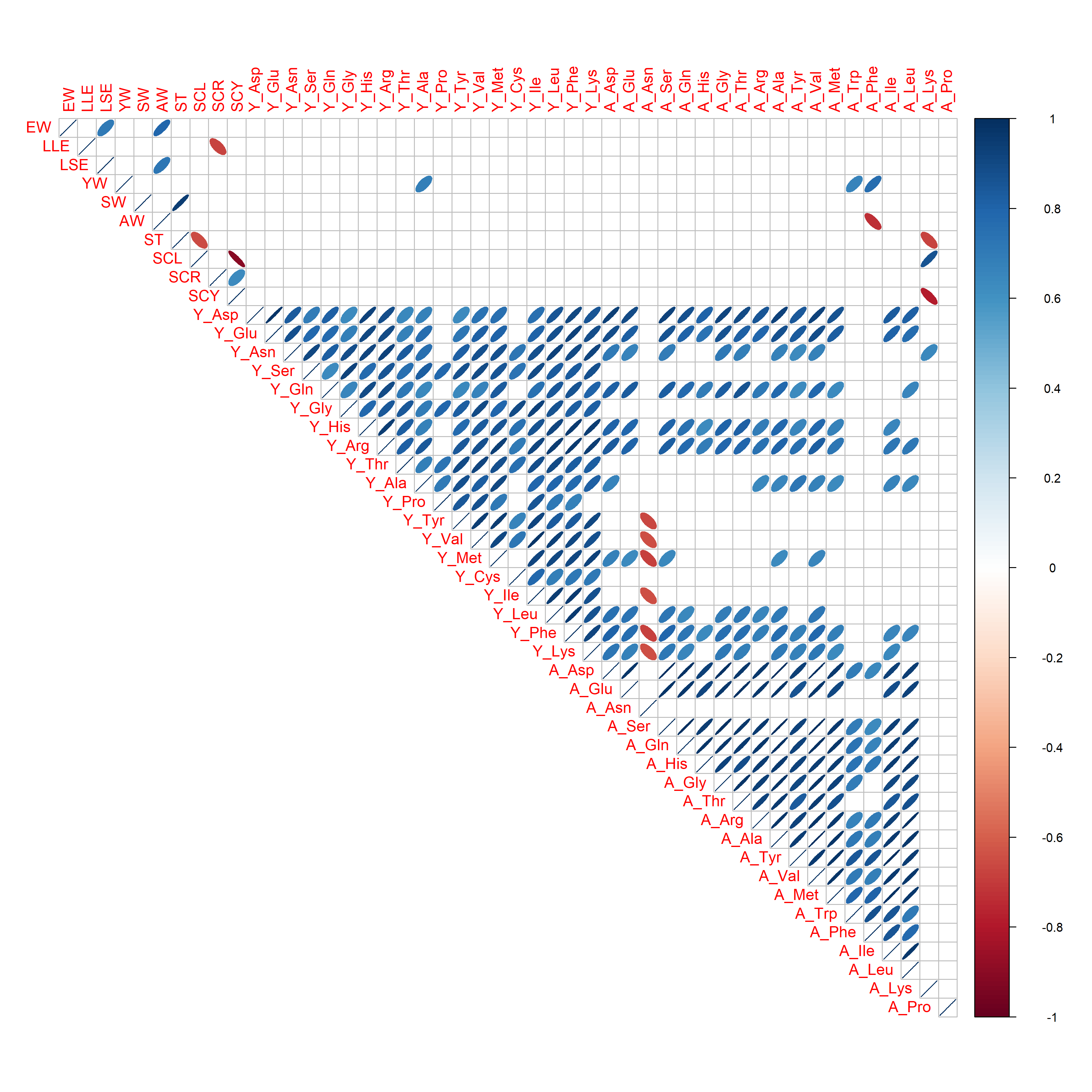

Supplement: S1 Fig — Ten egg traits, 19 yolk amino acids traits, and 19 albumen amino acids traits from 10 hens (ARA) were used. Trait abbreviations are shown in Materials and Methods and Results. Pearson’s correlations are expressed by ellipses. Blue and red ellipses indicate positive and negative correlations in each pair (P < 0.05). Blank cells show no significance. (TIFF) [file pone.0258506.s001.tiff]

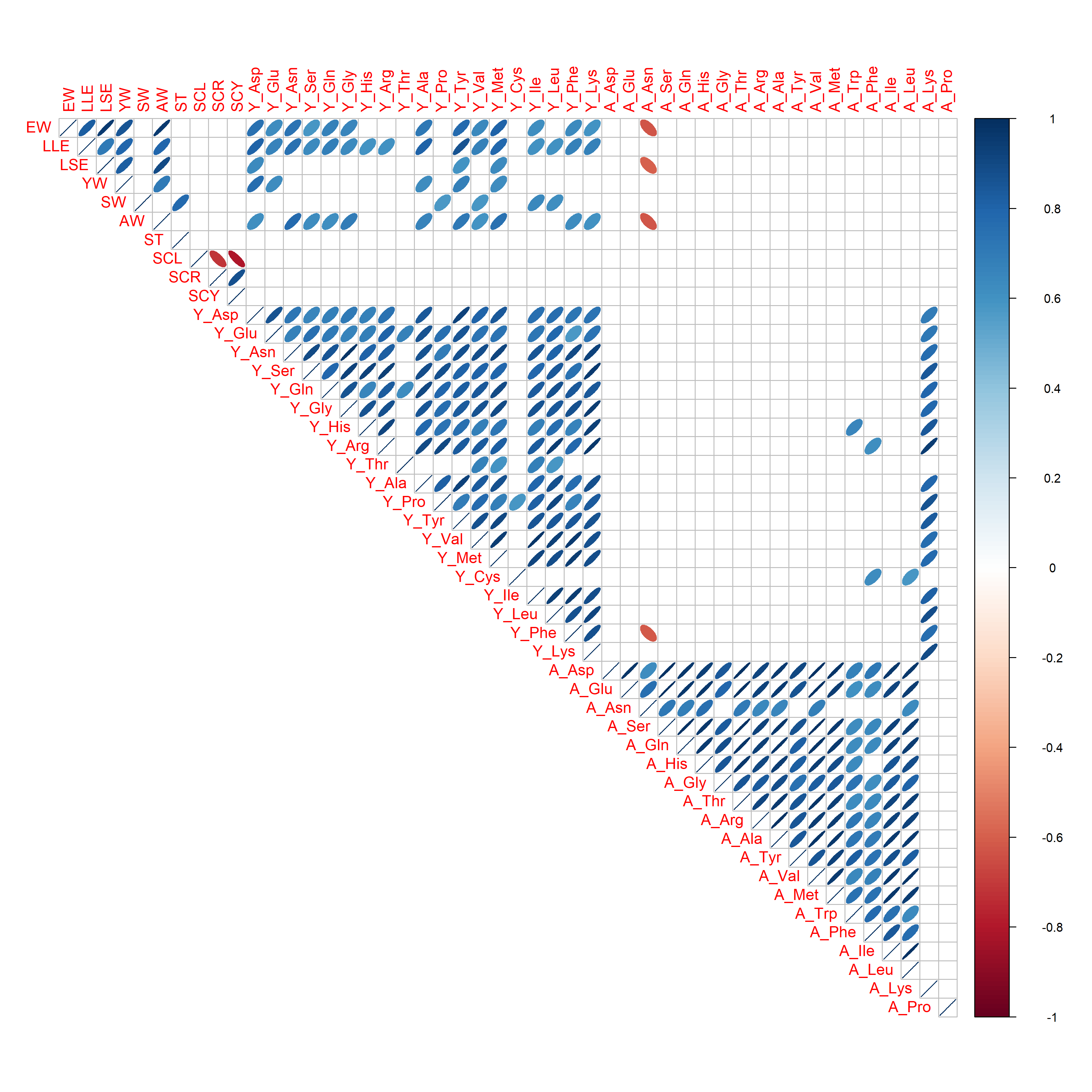

Supplement: S2 Fig — Ten egg traits, 19 yolk amino acids traits, and 19 albumen amino acids traits from 12 hens (KRH) were used. Trait abbreviations are shown in Materials and Methods and Results. Pearson’s correlations are expressed by ellipses. Blue and red ellipses indicate positive and negative correlations in each pair (P < 0.05). Blank cells show no significance. (TIFF) [file pone.0258506.s002.tiff]

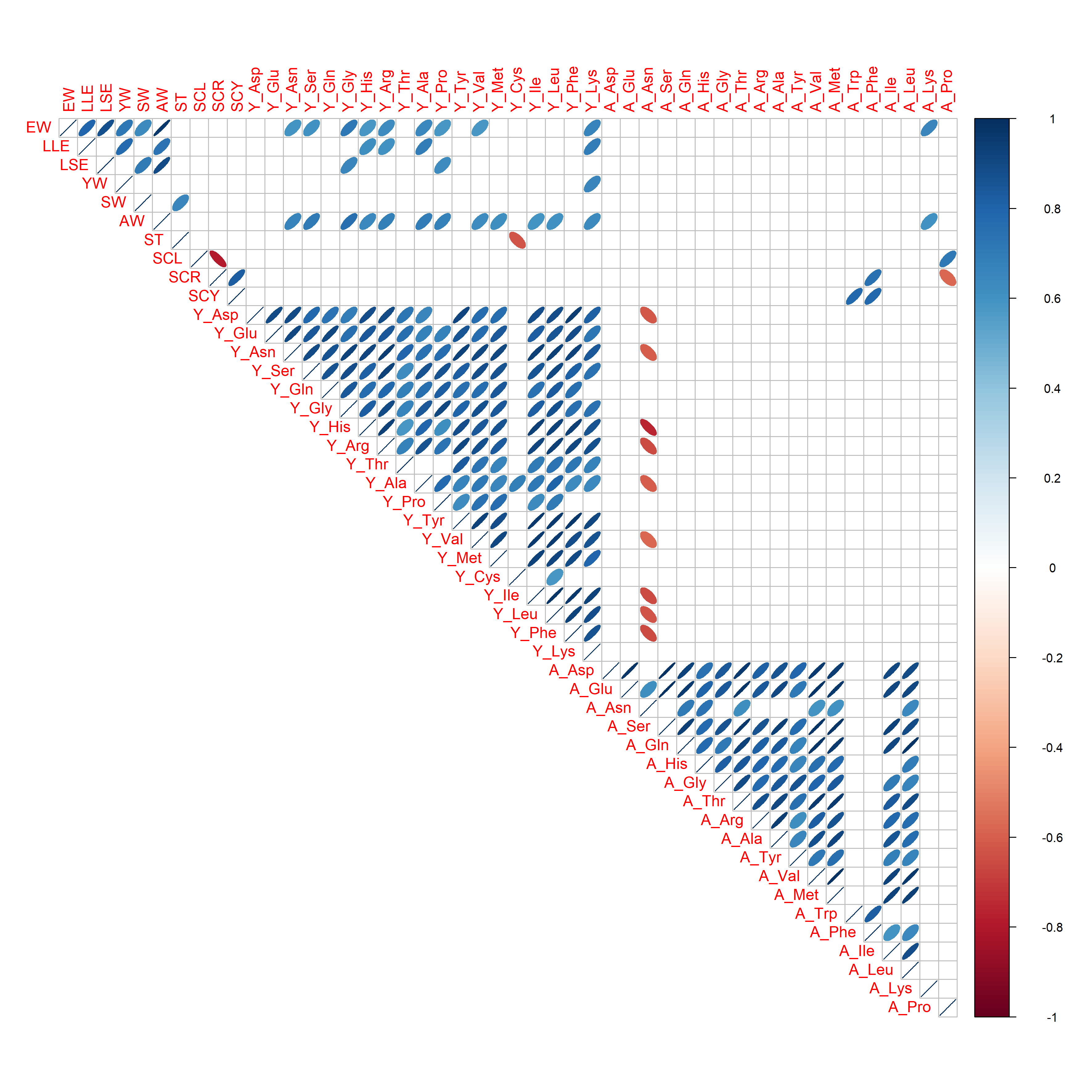

Supplement: S3 Fig — Ten egg traits, 19 yolk amino acids traits, and 19 albumen amino acids traits from 12 hens (UKO) were used. Trait abbreviations are shown in Materials and Methods and Results. Pearson’s correlations are expressed by ellipses. Blue and red ellipses indicate positive and negative correlations in each pair (P < 0.05). Blank cells show no significance. (TIFF) [file pone.0258506.s003.tiff]

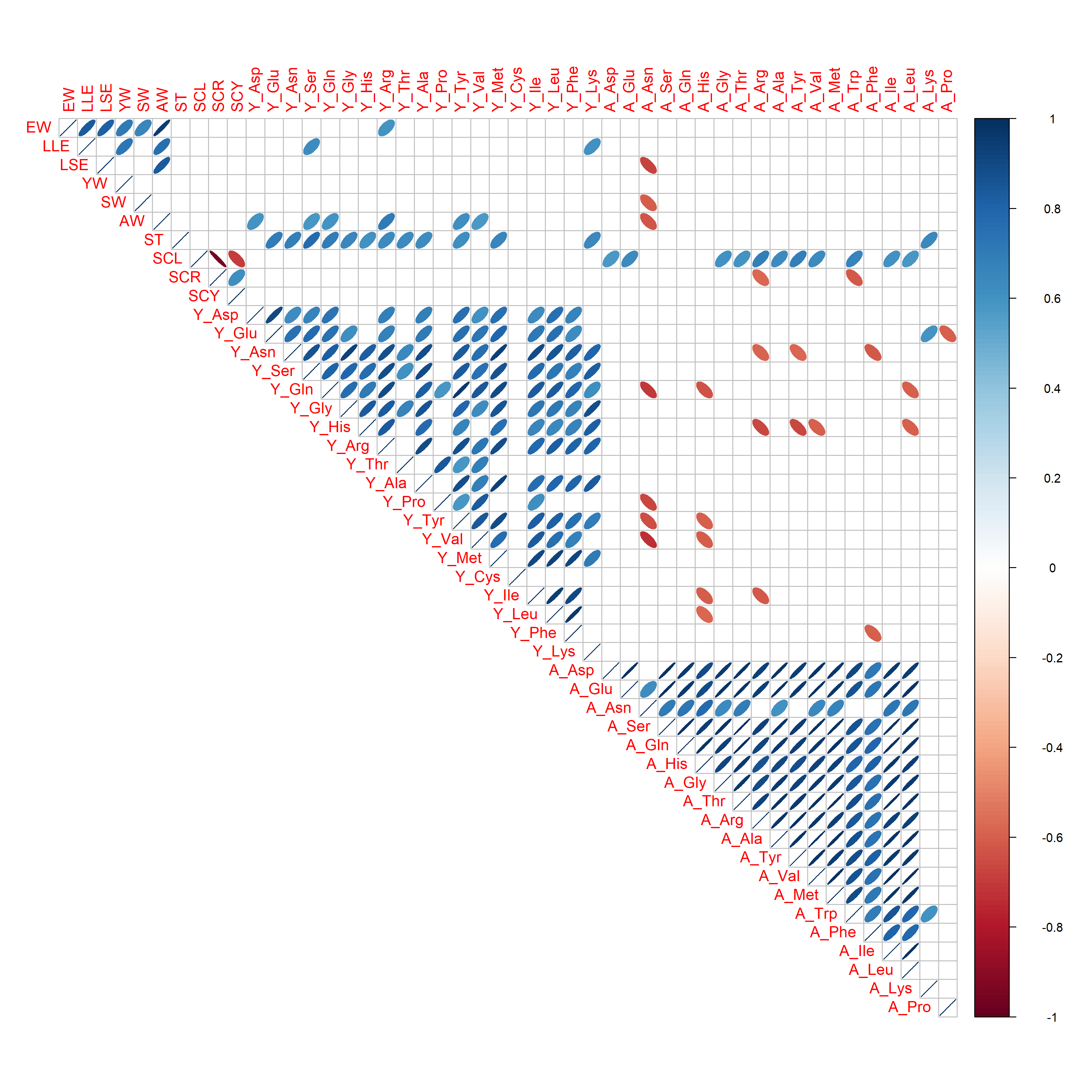

Supplement: S4 Fig — Ten egg traits, 19 yolk amino acids traits, and 19 albumen amino acids traits from 12 hens (NGY) were used. Trait abbreviations are shown in Materials and Methods and Results. Pearson’s correlations are expressed by ellipses. Blue and red ellipses indicate positive and negative correlations in each pair (P < 0.05). Blank cells show no significance. (TIFF) [file pone.0258506.s004.tiff]

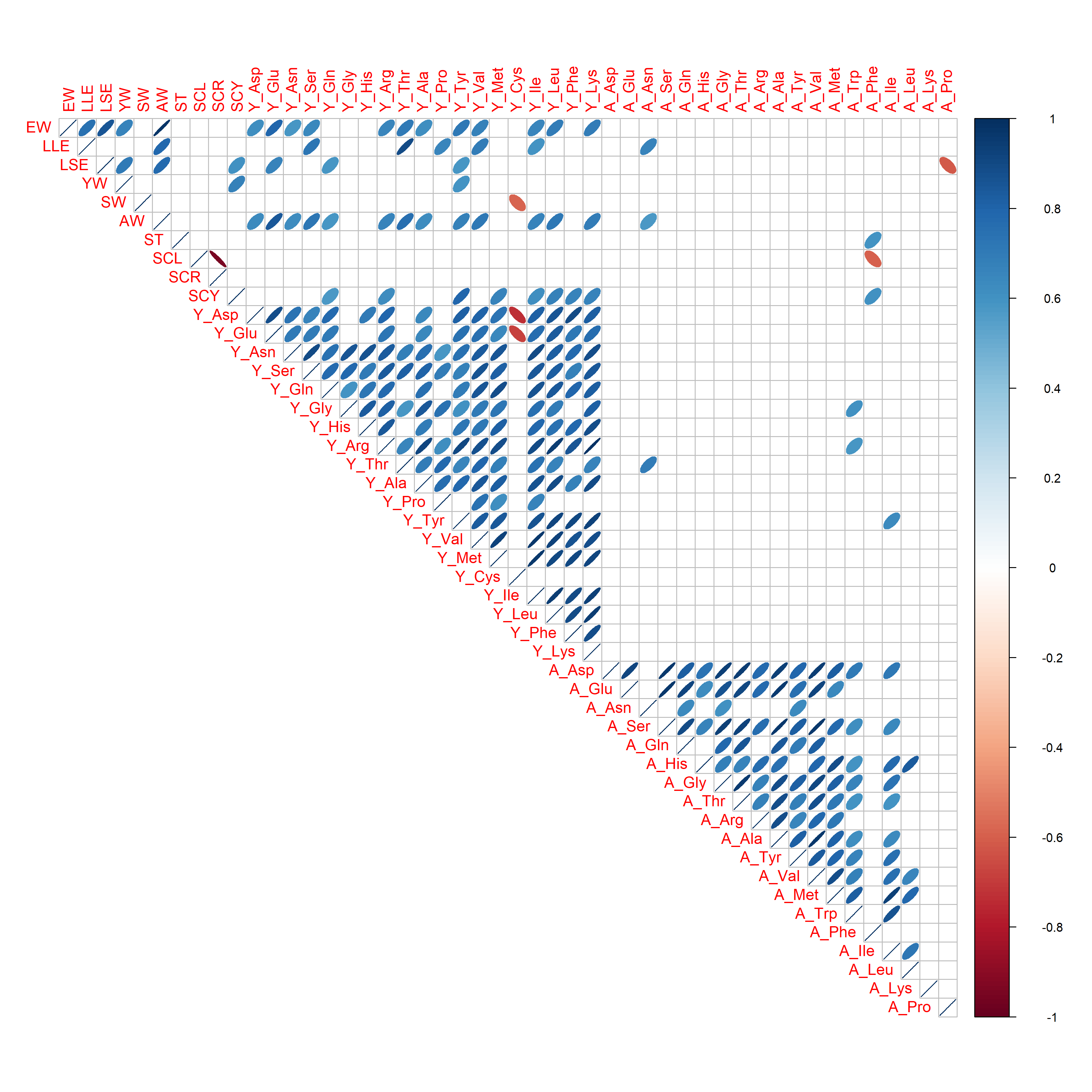

Supplement: S5 Fig — Ten egg traits, 19 yolk amino acids traits, and 19 albumen amino acids traits from 12 hens (BOR) were used. Trait abbreviations are shown in Materials and Methods and Results. Pearson’s correlations are expressed by ellipses. Blue and red ellipses indicate positive and negative correlations in each pair (P < 0.05). Blank cells show no significance. (TIFF) [file pone.0258506.s005.tiff]
