# Supplementary material for: Genetic effect on free amino acid contents of egg yolk and albumen using five different chicken genotypes under floor rearing system
Source: PLoS One. 2021 Oct 8;16(10):e0258506. doi: 10.1371/journal.pone.0258506 (PMC8500412; doi:10.1371/journal.pone.0258506)
Supplement: S1 Table — (DOCX) [file pone.0258506.s006.docx]

| **S1 Table**. Degree of freedom, F value, and P value of one-way ANOVA. | | | | | |
| --- | --- | --- | --- | --- | --- |
| Trait^1^ | One-way ANOVA^2^ | | | | |
|  | df_bet_ | df_res_ | F value | P value | |
| EW (g) | 4 | 51 | 54.1 | <2.2E-16 | *** |
| LLE (mm) | 4 | 51 | 29.9 | 8.2E-13 | *** |
| LSE (mm) | 4 | 51 | 49.2 | <2.2E-16 | *** |
| YW (g) | 4 | 51 | 28.1 | 2.4E-12 | *** |
| SW (g) | 4 | 51 | 29.4 | 1.1E-12 | *** |
| AW (g) | 4 | 51 | 41.0 | 2.4E-15 | *** |
| ST (mm) | 4 | 51 | 4.5 | 0.0033 | *** |
| SCL | 4 | 51 | 38.8 | 6.7E-15 | *** |
| SCR | 4 | 51 | 132.1 | <2.2E-16 | *** |
| SCY | 4 | 51 | 18.8 | 1.5E-09 | *** |
| Y_Asp | 4 | 51 | 11.2 | 1.4E-06 | *** |
| Y_Glu | 4 | 51 | 4.0 | 0.0068 | ** |
| Y_Asn | 4 | 51 | 0.5 | 0.7404 | ^ns^ |
| Y_Ser | 4 | 51 | 2.8 | 0.0368 | * |
| Y_Gln | 4 | 51 | 1.9 | 0.1205 | ^ns^ |
| Y_Gly | 4 | 51 | 4.0 | 0.0069 | ** |
| Y_His | 4 | 51 | 2.5 | 0.0509 | ^ns^ |
| Y_Arg | 4 | 51 | 0.1 | 0.9767 | ^ns^ |
| Y_Thr | 4 | 51 | 5.0 | 0.0018 | ** |
| Y_Ala | 4 | 51 | 2.4 | 0.0641 | ^ns^ |
| Y_Pro | 4 | 51 | 1.2 | 0.3149 | ^ns^ |
| Y_Tyr | 4 | 51 | 3.2 | 0.0202 | * |
| Y_Val | 4 | 51 | 2.0 | 0.1033 | ^ns^ |
| Y_Met | 4 | 51 | 1.7 | 0.1643 | ^ns^ |
| Y_Cys | 4 | 51 | 4.8 | 0.0023 | ** |
| Y_Ile | 4 | 51 | 1.4 | 0.2480 | ^ns^ |
| **S1 Table** (continued) Y_Leu | 4 | 51 | 2.6 | 0.0497 | * |
| Y_Phe | 4 | 51 | 0.3 | 0.8916 | ^ns^ |
| Y_Lys | 4 | 51 | 0.6 | 0.6393 | ^ns^ |
| A_Asp | 4 | 51 | 3.9 | 0.0077 | ** |
| A_Glu | 4 | 51 | 3.5 | 0.0134 | * |
| A_Asn | 4 | 51 | 3.8 | 0.0086 | ** |
| A_Ser | 4 | 51 | 3.0 | 0.0258 | * |
| A_Gln | 4 | 51 | 3.1 | 0.0244 | * |
| A_His | 4 | 51 | 2.9 | 0.0324 | * |
| A_Gly | 4 | 51 | 1.6 | 0.1971 | ^ns^ |
| A_Thr | 4 | 51 | 2.2 | 0.0879 | ^ns^ |
| A_Arg | 4 | 51 | 1.8 | 0.1462 | ^ns^ |
| A_Ala | 4 | 51 | 2.7 | 0.0416 | * |
| A_Tyr | 4 | 51 | 3.7 | 0.0098 | ** |
| A_Val | 4 | 51 | 2.3 | 0.0715 | ^ns^ |
| A_Met | 4 | 51 | 2.3 | 0.0668 | ^ns^ |
| A_Trp | 4 | 51 | 7.1 | 0.0001 | *** |
| A_Phe | 4 | 51 | 5.0 | 0.0019 | ** |
| A_Ile | 4 | 51 | 2.9 | 0.0313 | * |
| A_Leu | 4 | 51 | 2.0 | 0.1029 | ^ns^ |
| A_Lys | 4 | 51 | 0.6 | 0.6326 | ^ns^ |
| A_Pro | 4 | 51 | 0.3 | 0.8918 | ^ns^ |
| ^1^ Trait abbreviations are shown in Materials and Methods. | | | | | |
| ^2^ df_bet_: between groups degree of freedom, df_res_: residual degree of freedom, *** P < 0.001, ** P < 0.01, * P < 0.05, ^ns^ P > 0.05. | | | | | |
