# Supplementary material for: Genetic effect on free amino acid contents of egg yolk and albumen using five different chicken genotypes under floor rearing system
Source: PLoS One. 2021 Oct 8;16(10):e0258506. doi: 10.1371/journal.pone.0258506 (PMC8500412; doi:10.1371/journal.pone.0258506)
Supplement: Attachment — Submitted filename: Nishimura_et_al_Main.docx [file pone.0258506.s008.docx]

**Genetic effect on free amino acid contents of egg yolk and albumen using five different chicken genotypes under floor rearing system**

Kenji Nishimura^1^, Daichi Ijiri^2^, Saki Shimamoto^2,3^, Masahiro Takaya^1,4^, Akira Ohtsuka^2^, Tatsuhiko Goto^1,5 *^

^1^ Department of Life and Food Science, Obihiro University of Agriculture and Veterinary Medicine, Obihiro, Hokkaido 080-8555, Japan

^2^ Department of Biochemical Science and Technology, Kagoshima University, Korimoto, Kagoshima 890-0065, Japan

^3^ Graduate School of Science and Technology, Niigata University, Niigata 950-2181, Japan

^4^ Hokkaido Tokachi Area Regional Food Processing Technology Center, Tokachi Foundation 080-2462, Japan

^5^ Research Center for Global Agromedicine, Obihiro University of Agriculture and Veterinary Medicine, Obihiro, Hokkaido 080-8555, Japan

*** Corresponding author**

Email: tats.goto@obihiro.ac.jp (TG)

**Abstract**

Chicken eggs play an important role as food resources in the world. Although genetic effects on yolk and albumen contents have been reported, the number of chicken genotypes analyzed so far is still limited. To investigate the effect of genetic background on 10 egg traits, 19 yolk amino acid traits, and 19 albumen amino acid traits, we evaluated a total of 58 eggs from five genotypes: Araucana (ARA), Kurohisui (KRH), Ukokkei (UKO), Nagoya (NGY), and Boris Brown (BOR) under a free-range floor rearing system. One-way ANOVA revealed significant effects of genotype on 10 egg traits, 8 yolk amino acids (Asp, Glu, Ser, Gly, Thr, Tyr, Cys, and Leu), and 11 albumen amino acids (Asp, Glu, Asn, Ser, Gln, His, Ala, Tyr, Trp, Phe, and Ile) contents. Moderate to strong positive phenotypic correlations among traits within each trait category (size and weight traits, yolk amino acid traits, and albumen amino acid traits), whereas there were basically no or weak correlations among the trait categories. However, a unique feature was found in ARA indicating moderate positive correlations of amino acids between yolk and albumen. These results suggest that genetic factors can modify not only size, weight, and color of the egg but also yolk and albumen free amino acids contents.

**Introduction**

Chicken is likely to be domesticated from red jungle fowls in southwestern China, Thailand, and Myanmar around 9500 ± 3300 years ago [1]. The number of chickens exceeded 22 billion in 2017, while there are approximately 1600 local chicken breeds around the world [2]. There are many kinds of chickens that have extreme phenotypic characteristics such as body size, feathers, growth, eggs, meat, and behavior. For example, the Japanese indigenous chicken breeds of “Onagadori” has extremely long tail feathers in male [3, 4], while the weight of male “Oh-Shamo” reaches 4-6 kg [3, 6, 7]. Thus, the indigenous chickens have large potential for studying genetic and phenotypic diversity [8].

Eggs are very useful sources of high-quality protein in developed and developing countries alike [8]. From the first half of the 20th century, specialized breeding towards high egg production were started in order to increase the number of eggs. As a result of the breeding using phenotypic and pedigree information, recent chicken lines express high egg production with peak egg laying rates of 95-97% [9]. However, the commercial lines based on White Leghorn, White Plymouth Rock, and Rhode Island Red in the layer industry are only a handful of breeds as opposed to indigenous breeds of chickens in the world. To improve the existing egg quality traits, further investigating these genetic resources may be important.

Egg traits such as egg weight, eggshell color, yolk size, albumen height [10-13], and ingredients of yolk and albumen [14-16] are fairly influenced by genetic factors. Therefore, eggs from several breeds may express a large variation in the appearance of egg and contents of yolk and albumen caused by differences in genetic background. For example, eggshell color of the Araucana that has South American origin is blue, which is significantly different from the white eggshell color of White Leghorn [17]. Also, it has been reported that contents of fatty acids, cholesterol, protein, ash, and mineral in eggs [18-20] significantly differed among several breeds and strains. Significant effects of breed differences on free amino acids of yolk and albumen have been reported [16, 21-23]. Free amino acids are characterized by acting on taste as well as nutritional components [24].

The relationship between amino acids and taste (sweetness, sourness, saltiness, bitterness, and umami) has been studied. It is known that amino acids induce the response of taste related sensory cells using a mouse model [25]. Glutamic acid has the highest effect towards umami among amino acids [26], whereas serine is known to be related to sweetness [24]. Goto et al. [23] reported significant breed and feed effects on amino acids and taste sensor traits of albumen and yolk using Australorp and Rhode Island Red fed mixed and fermented feeds. Although genetic impact has been shown on free amino acids of yolk and albumen using Australorp, Nagoya (NGY), Rhode Island Red (RIR), Shamo (SHA), Ukokkei, and two F_1_ hybrids (NGY × RIR and SHA × RIR) [16], the number of breeds analyzed is still limited. Moreover, these studies have been conducted under cage rearing system. Therefore, we hypothesized that there are some different features in free amino acid contents of egg yolk and albumen using untapped several chicken genotypes under floor rearing system. The purpose of this study was to investigate effect of genetic background on egg traits and free amino acid contents of yolk and albumen using five genotypes (Araucana, Kurohisui, Ukokkei, Nagoya, and Boris Brown) under free-range floor rearing system.

**Materials and methods**

This study was approved from the Experimental Animal Committee of the Obihiro University of Agriculture and Veterinary Medicine (authorization number 18–15).

**Chickens**

A total of 58 eggs obtained from adult hens (72 weeks of age) from Araucana cross (ARA; n = 10), Kurohisui (KRH; n = 12), Ukokkei (UKO; n = 12), Nagoya (NGY; n = 12), and a Brown layer (Boris Brown (BOR); n = 12) were used. Five genotypes of chickens in the Tago Poultry Farm (Obihiro, Japan) were kept under a free-range rearing system. Since we collected all eggs in a day of July, total of 58 hens were used in this study. The Tago Poultry Farm uses originally blended feed for all the genotypes. The 20 kg of mixed feed for layers ([Chubu](https://eow.alc.co.jp/search?q=Chubu&ref=awlj) [Shiryo](https://eow.alc.co.jp/search?q=Shiryo&ref=awlj) Co., Ltd., Japan) was mixed with 10 kg of crushed corn, 10 kg of rice bran, 7 kg of beet pulp, 0.5 kg of scallop shell, and 0.5 kg of fish meal.

**Measurement of egg traits**

We measured a total of 10 egg traits including egg weight (EW), yolk weight (YW), albumen weight (AW), eggshell weight (SW), length of the long axis of egg (LLE), length of the short axis of egg (LSE), eggshell thickness (ST), eggshell color lightness (SCL), eggshell color redness (SCR), and eggshell color yellowness (SCY). Egg weight, yolk weight, and eggshell weight were measured by an electronic balance (EK-6000H; A&D Company, Ltd., Japan), whereas albumen weight was obtained by subtracting weights of yolk and eggshell from egg weight. Lengths of the long and short axes of egg were measured using a digital caliper (P01 110–120; ASONE, Japan), while a Peacock dial pipe gauge (P-1; Ozaki MFG Co., Ltd., Japan) was used to measure the eggshell thickness. Eggshell colors were measured by a chromameter (CR-10 Plus Color Reader; Konica Minolta Japan, Inc., Japan), which confirmed that breeders can use Minolta colorimetry with confidence to assess differences in staining to estimate cuticle deposition (absorbance at 640 nm) [11]. After measuring the 10 egg traits, the yolk was diluted 2-fold with distilled water (DW) for free amino acid analysis. Then, the yolk solution and albumen were stored at -30°C until use.

**Free amino acid analysis of yolk**

The 5 mL of 2-fold diluted yolk solution was thawed with lukewarm water. Then, 5 mL of 16% trichloroacetic acid solution (FUJIFILM Wako Chemicals, Japan) was added to each sample and mixed thoroughly by vortex. The mixture was centrifuged at 1,400 × g for 15 minutes using a table-top centrifuge (model 2410; KUBOTA Corporation Co., Ltd., Japan) or a centrifuge (RX II series; HITACHI Ltd., Japan). The supernatant was filtered using a syringe (NIPRO Corporation, Japan) and a 0.45 µm pore size membrane filter (DISMIC-25CS; Advantec Toyo Kaisha, Ltd., Japan). Then, 40 µL of the filtered sample was dried at 40°C for 90 min using a vacuum constant temperature dryer (VOS-201SD, Eyela, Japan). After adding 20 µL of mixing solution (ethanol: DW: triethylamine = 2:2:1), the tube was mixed for 20 min using a Micro Mixer E-36 (TAITEC Corporation, Japan). The sample was heated at 40°C for 60 min in vacuum to dry. After adding 20 µL of the mixed solution (ethanol: DW: triethylamine: phenylisothiocyanate = 7:1:1:1), the tube was vortexed for 20 min. After vacuum drying at 40°C for 90 min, the sample was kept at -30°C until the amino acid analysis.

Detection of free amino acids in yolk was performed by HPLC (LC-2010CHT; Shimadzu Co. Ltd., Japan). Amino acid standards (B-type and H-type), L-asparagine, and L-glutamine were prepared in the same manner as the sample pretreatment. The concentration of free amino acid in yolk was determined from the peaks of the sample and standards [16].

**Free amino acid analysis of albumen**

The frozen albumen sample was thawed and mixed thoroughly. Incubation was carried out at room temperature for 3 min to separate parts of the foam and liquid. The 500 μL of albumen sample, 500 μL of MilliQ water, and 1,000 μL of acetonitrile were mixed (albumen sample: water: acetonitrile = 1: 1: 2) and vortexed for 1 min. Then, centrifugation was performed at 22,000 × g for 5 min, and the lower layer was filtered with a 0.2 µm pore sized filter.

The free amino acids of albumen sample were analyzed by UHPLC (NexeraX2, Shimadzu 353 Co., Ltd., Japan) using Kinetex 2.6 μm (EVO C18 100 × 3.0 mm) for the column (Goto et al., 2021b). Gradient release was used in this study with mobile phase A (17 mM potassium dihydrogen phosphate and 3 mM dipotassium hydrogen phosphate) and mobile phase B (water:acetonitrile:methanol = 15:45:40). For pre-column derivatization, 45 μL of mercaptopropionic acid, 22 μL of ortho-phthalaldehyde, and 7.5 μL of albumen sample were mixed and incubated at room temperature for 1 min. Then, 5 μL of fluorenylmethyl chloroformate was mixed with the solution and incubated at room temperature for 2 min. The 1 μL of the derivatized solution was injected into the column at a flow rate of 0.85 mL/min. A fluorescence detector RF-20AXS was used for wavelength detection. In this study, wavelengths of Ch1 EX 350 nm EM 450 nm and Ch2 EX 266 nm EM 305 nm were used [16].

**Statistical analysis**

A one-way analysis of variance (ANOVA) with a significant threshold (P < 0.05) was performed to test effect on genotype in egg traits and amino acids traits. Tukey’s HSD test with a significant threshold (P < 0.05) was performed to see difference in each pair of genotypes. Data were presented as mean ± standard deviation.

Pearson’s correlation was estimated among all traits using ‘corrplot’ package in R [27]. Significant threshold (P < 0.05) was used to express whether correlation coefficients equal zero. All statistical analyses were performed using R.

**Results**

**Egg traits**

Egg traits in five genotypes (ARA, KRH, UKO, NGY, and BOR) are indicated in Table 1 and Fig. S1. In 10 traits (EW, YW, AW, SW, LLE, LSE, ST, SCL, SCR, and SCY), significant effects of genotype were observed (P < 0.05). Egg weight, albumen weight, and eggshell weight of BOR were the highest among five genotypes. On the other hand, yolk weight tended to be higher in ARA, KRH, and NGY than BOR. Egg weight, albumen weight, yolk weight, eggshell weight, LLE, and LSE were lower in UKO than others. Eggshell redness was the highest in BOR (16.3 ± 1.9) and lowest in ARA (-2.6 ± 2.3).

| **Table 1**. Mean and standard deviation of egg traits in five genotypes of chickens | | | | | | | | | | | | | | | | | | | | | | | | | |
| --- | --- | --- | --- | --- | --- | --- | --- | --- | --- | --- | --- | --- | --- | --- | --- | --- | --- | --- | --- | --- | --- | --- | --- | --- | --- |
| Trait^1^ | Araucana Cross | | |  | Kurohisui | | |  | Ukokkei | | |  | Nagoya | | |  | Boris Brown | | |  | One-way ANOVA^2^ | | | | |
|  | (ARA; *n* = 10) | | |  | (KRH; *n* = 12) | | |  | (UKO; *n* = 12) | | |  | (NGY; *n* = 12) | | |  | (BOR; *n* = 12) | | |  | df_bet_ | df_res_ | F | P | |
| EW (g) | 56.7 | ± | 3.0 |  | 54.3 | ± | 5.2 |  | 39.4 | ± | 2.6 |  | 56.7 | ± | 4.3 |  | 61.6 | ± | 3.6 |  | 4 | 51 | 54.1 | < 2.2E-16 | *** |
| LLE (mm) | 55.3 | ± | 1.6 |  | 55.8 | ± | 2.8 |  | 49.3 | ± | 1.6 |  | 55.5 | ± | 1.8 |  | 58.1 | ± | 2.1 |  | 4 | 51 | 29.9 | 8.2E-13 | *** |
| LSE (mm) | 43.0 | ± | 1.3 |  | 41.7 | ± | 1.2 |  | 37.8 | ± | 0.9 |  | 42.7 | ± | 1.2 |  | 43.7 | ± | 0.9 |  | 4 | 51 | 49.2 | < 2.2E-16 | *** |
| YW (g) | 16.7 | ± | 1.8 |  | 17.2 | ± | 1.8 |  | 12.5 | ± | 0.6 |  | 17.5 | ± | 1.2 |  | 16.6 | ± | 1.0 |  | 4 | 51 | 28.1 | 2.4E-12 | *** |
| SW (g) | 6.9 | ± | 0.6 |  | 6.9 | ± | 1.0 |  | 4.8 | ± | 0.4 |  | 6.4 | ± | 0.8 |  | 7.6 | ± | 0.5 |  | 4 | 51 | 29.4 | 1.1E-12 | *** |
| AW (g) | 33.1 | ± | 3.4 |  | 30.3 | ± | 3.4 |  | 22.1 | ± | 1.9 |  | 32.8 | ± | 3.1 |  | 37.4 | ± | 2.8 |  | 4 | 51 | 41.0 | 2.4E-15 | *** |
| ST (mm) | 0.44 | ± | 0.04 |  | 0.42 | ± | 0.04 |  | 0.38 | ± | 0.03 |  | 0.39 | ± | 0.04 |  | 0.42 | ± | 0.04 |  | 4 | 51 | 4.5 | 0.0033 | *** |
| SCL | 74.6 | ± | 4.6 |  | 64.1 | ± | 2.8 |  | 73.5 | ± | 3.2 |  | 72.8 | ± | 4.5 |  | 57.4 | ± | 3.7 |  | 4 | 51 | 38.8 | 6.7E-15 | *** |
| SCR | -2.6 | ± | 2.2 |  | 0.7 | ± | 2.1 |  | 8.3 | ± | 1.9 |  | 9.1 | ± | 2.5 |  | 16.3 | ± | 1.9 |  | 4 | 51 | 132.1 | < 2.2E-16 | *** |
| SCY | 15.5 | ± | 4.2 |  | 21.4 | ± | 2.7 |  | 19.9 | ± | 1.9 |  | 18.4 | ± | 2.5 |  | 25.2 | ± | 1.3 |  | 4 | 51 | 18.8 | 1.5E-09 | *** |
| Y_Asp | 27.6 | ± | 7.0 |  | 42.9 | ± | 12.7 |  | 32.1 | ± | 8.9 |  | 55.7 | ± | 13.9 |  | 33.8 | ± | 9.3 |  | 4 | 51 | 11.2 | 1.4E-06 | *** |
| Y_Glu | 166.6 | ± | 19.4 |  | 206.9 | ± | 27.5 |  | 181.5 | ± | 20.7 |  | 183.1 | ± | 21.6 |  | 173.8 | ± | 18.7 |  | 4 | 51 | 4.0 | 0.0068 | ** |
| Y_Asn | 35.0 | ± | 4.5 |  | 34.8 | ± | 4.5 |  | 33.3 | ± | 4.0 |  | 34.5 | ± | 3.4 |  | 35.6 | ± | 3.1 |  | 4 | 51 | 0.5 | 0.7404 | ^ns^ |
| Y_Ser | 57.9 | ± | 6.1 |  | 59.9 | ± | 5.6 |  | 54.2 | ± | 4.9 |  | 61.2 | ± | 5.0 |  | 59.7 | ± | 4.9 |  | 4 | 51 | 2.8 | 0.0368 | * |
| Y_Gln | 66.5 | ± | 4.5 |  | 73.1 | ± | 6.5 |  | 67.1 | ± | 5.5 |  | 66.2 | ± | 5.9 |  | 69.2 | ± | 5.0 |  | 4 | 51 | 1.9 | 0.1205 | ^ns^ |
| Y_Gly | 22.2 | ± | 2.4 |  | 19.6 | ± | 2.5 |  | 19.9 | ± | 1.8 |  | 22.0 | ± | 2.1 |  | 22.0 | ± | 1.9 |  | 4 | 51 | 4.0 | 0.0069 | ** |
| Y_His | 17.6 | ± | 3.1 |  | 16.6 | ± | 2.9 |  | 21.3 | ± | 5.5 |  | 18.3 | ± | 2.2 |  | 18.8 | ± | 3.4 |  | 4 | 51 | 2.5 | 0.0509 | ^ns^ |
| Y_Arg | 75.4 | ± | 10.5 |  | 78.0 | ± | 8.4 |  | 76.2 | ± | 10.7 |  | 76.1 | ± | 5.4 |  | 77.4 | ± | 7.9 |  | 4 | 51 | 0.1 | 0.9767 | ^ns^ |
| Y_Thr | 61.0 | ± | 6.6 |  | 71.3 | ± | 9.8 |  | 61.6 | ± | 5.3 |  | 60.0 | ± | 4.7 |  | 60.0 | ± | 3.7 |  | 4 | 51 | 5.0 | 0.0018 | ** |
| Y_Ala | 37.9 | ± | 4.2 |  | 43.9 | ± | 4.3 |  | 40.2 | ± | 4.1 |  | 40.9 | ± | 3.5 |  | 39.8 | ± | 3.4 |  | 4 | 51 | 2.4 | 0.0641 | ^ns^ |
| Y_Pro | 41.3 | ± | 3.4 |  | 40.2 | ± | 2.9 |  | 39.1 | ± | 2.4 |  | 39.3 | ± | 2.9 |  | 41.0 | ± | 2.6 |  | 4 | 51 | 1.2 | 0.3149 | ^ns^ |
| Y_Tyr | 75.2 | ± | 7.6 |  | 70.3 | ± | 6.1 |  | 67.1 | ± | 6.4 |  | 74.3 | ± | 5.4 |  | 69.8 | ± | 5.4 |  | 4 | 51 | 3.2 | 0.0202 | * |
| Y_Val | 68.7 | ± | 7.4 |  | 68.3 | ± | 6.3 |  | 62.8 | ± | 5.8 |  | 63.4 | ± | 4.8 |  | 66.3 | ± | 4.4 |  | 4 | 51 | 2.0 | 0.1033 | ^ns^ |
| **Table 1 (continued)**  Y_Met | 28.7 | ± | 3.1 |  | 29.9 | ± | 3.5 |  | 27.3 | ± | 3.2 |  | 30.3 | ± | 3.0 |  | 30.3 | ± | 2.5 |  | 4 | 51 | 1.7 | 0.1643 | ^ns^ |
| Y_Cys | 2.8 | ± | 0.4 |  | 2.7 | ± | 0.5 |  | 2.5 | ± | 0.9 |  | 1.8 | ± | 0.6 |  | 2.4 | ± | 0.5 |  | 4 | 51 | 4.8 | 0.0023 | ** |
| Y_Ile | 64.1 | ± | 6.4 |  | 62.7 | ± | 6.3 |  | 59.2 | ± | 5.8 |  | 63.6 | ± | 4.4 |  | 60.8 | ± | 4.1 |  | 4 | 51 | 1.4 | 0.2480 | ^ns^ |
| Y_Leu | 120.9 | ± | 13.1 |  | 125.3 | ± | 12.7 |  | 113.4 | ± | 12.4 |  | 112.2 | ± | 8.7 |  | 122.6 | ± | 9.4 |  | 4 | 51 | 2.6 | 0.0497 | * |
| Y_Phe | 55.9 | ± | 7.6 |  | 55.2 | ± | 6.8 |  | 53.2 | ± | 7.2 |  | 53.3 | ± | 5.3 |  | 54.1 | ± | 5.6 |  | 4 | 51 | 0.3 | 0.8916 | ^ns^ |
| Y_Lys | 92.0 | ± | 13.2 |  | 92.9 | ± | 10.4 |  | 92.2 | ± | 11.7 |  | 96.5 | ± | 8.5 |  | 89.3 | ± | 8.8 |  | 4 | 51 | 0.6 | 0.6393 | ^ns^ |
| A_Asp | 0.57 | ± | 0.28 |  | 0.54 | ± | 0.19 |  | 0.57 | ± | 0.16 |  | 0.93 | ± | 0.49 |  | 0.52 | ± | 0.10 |  | 4 | 51 | 3.9 | 0.0077 | ** |
| A_Glu | 1.00 | ± | 0.38 |  | 0.87 | ± | 0.27 |  | 1.06 | ± | 0.28 |  | 1.44 | ± | 0.74 |  | 0.82 | ± | 0.12 |  | 4 | 51 | 3.5 | 0.0134 | * |
| A_Asn | 0.03 | ± | 0.01 |  | 0.02 | ± | 0.01 |  | 0.04 | ± | 0.02 |  | 0.09 | ± | 0.11 |  | 0.02 | ± | 0.01 |  | 4 | 51 | 3.8 | 0.0086 | ** |
| A_Ser | 0.57 | ± | 0.28 |  | 0.49 | ± | 0.21 |  | 0.57 | ± | 0.17 |  | 0.85 | ± | 0.53 |  | 0.43 | ± | 0.10 |  | 4 | 51 | 3.0 | 0.0258 | * |
| A_Gln | 1.18 | ± | 0.51 |  | 1.07 | ± | 0.55 |  | 1.15 | ± | 0.36 |  | 1.97 | ± | 1.50 |  | 0.84 | ± | 0.28 |  | 4 | 51 | 3.1 | 0.0244 | * |
| A_His | 0.18 | ± | 0.07 |  | 0.13 | ± | 0.07 |  | 0.23 | ± | 0.06 |  | 0.23 | ± | 0.13 |  | 0.16 | ± | 0.04 |  | 4 | 51 | 2.9 | 0.0324 | * |
| A_Gly | 0.12 | ± | 0.06 |  | 0.10 | ± | 0.05 |  | 0.11 | ± | 0.03 |  | 0.15 | ± | 0.08 |  | 0.10 | ± | 0.04 |  | 4 | 51 | 1.6 | 0.1971 | ^ns^ |
| A_Thr | 0.52 | ± | 0.27 |  | 0.45 | ± | 0.19 |  | 0.51 | ± | 0.18 |  | 0.69 | ± | 0.41 |  | 0.38 | ± | 0.10 |  | 4 | 51 | 2.2 | 0.0879 | ^ns^ |
| A_Arg | 0.70 | ± | 0.29 |  | 0.60 | ± | 0.19 |  | 0.69 | ± | 0.21 |  | 0.87 | ± | 0.43 |  | 0.59 | ± | 0.11 |  | 4 | 51 | 1.8 | 0.1462 | ^ns^ |
| A_Ala | 0.34 | ± | 0.19 |  | 0.31 | ± | 0.14 |  | 0.39 | ± | 0.13 |  | 0.50 | ± | 0.31 |  | 0.24 | ± | 0.07 |  | 4 | 51 | 2.7 | 0.0416 | * |
| A_Tyr | 0.75 | ± | 0.26 |  | 0.77 | ± | 0.15 |  | 0.89 | ± | 0.17 |  | 1.05 | ± | 0.45 |  | 0.64 | ± | 0.13 |  | 4 | 51 | 3.7 | 0.0098 | ** |
| A_Val | 0.61 | ± | 0.30 |  | 0.51 | ± | 0.22 |  | 0.66 | ± | 0.16 |  | 0.76 | ± | 0.41 |  | 0.45 | ± | 0.10 |  | 4 | 51 | 2.3 | 0.0715 | ^ns^ |
| A_Met | 0.49 | ± | 0.23 |  | 0.38 | ± | 0.17 |  | 0.54 | ± | 0.10 |  | 0.58 | ± | 0.30 |  | 0.37 | ± | 0.10 |  | 4 | 51 | 2.3 | 0.0668 | ^ns^ |
| A_Trp | 0.28 | ± | 0.08 |  | 0.32 | ± | 0.04 |  | 0.35 | ± | 0.04 |  | 0.35 | ± | 0.06 |  | 0.24 | ± | 0.05 |  | 4 | 51 | 7.1 | 0.0001 | *** |
| A_Phe | 1.22 | ± | 0.20 |  | 1.25 | ± | 0.21 |  | 1.53 | ± | 0.16 |  | 1.31 | ± | 0.22 |  | 1.24 | ± | 0.10 |  | 4 | 51 | 5.0 | 0.0019 | ** |
| A_Ile | 0.58 | ± | 0.21 |  | 0.51 | ± | 0.15 |  | 0.68 | ± | 0.08 |  | 0.68 | ± | 0.25 |  | 0.49 | ± | 0.08 |  | 4 | 51 | 2.9 | 0.0313 | * |
| A_Leu | 1.39 | ± | 0.38 |  | 1.16 | ± | 0.31 |  | 1.50 | ± | 0.15 |  | 1.47 | ± | 0.50 |  | 1.19 | ± | 0.17 |  | 4 | 51 | 2.0 | 0.1029 | ^ns^ |
| A_Lys | 0.03 | ± | 0.02 |  | 0.03 | ± | 0.02 |  | 0.04 | ± | 0.02 |  | 0.05 | ± | 0.02 |  | 0.04 | ± | 0.04 |  | 4 | 51 | 0.6 | 0.6326 | ^ns^ |
| A_Pro | 0.64 | ± | 0.11 |  | 0.66 | ± | 0.10 |  | 0.65 | ± | 0.08 |  | 0.62 | ± | 0.13 |  | 0.68 | ± | 0.18 |  | 4 | 51 | 0.3 | 0.8918 | ^ns^ |
| ^1^ Trait abbreviations are shown in Materials and Methods and Results. Amino acids concentrations are shown in µg/mL. | | | | | | | | | | | | | | | | | | | | | | | | | |
| **Table 1 (continued)**  ^2^ df_bet_: between groups degree of freedom, df_res_: residual degree of freedom, *** P < 0.001, ** P < 0.01, * P < 0.05, ^ns^ P > 0.05. | | | | | | | | | | | | | | | | | | | | | | | | | |
| Results of Tukey’s HSD tests were shown in Figures S1,S2, and S3. | | | | | | | | | | | | | | | | | | | | | | | | | |

**Yolk free amino acids**

This study detected 19 yolk free amino acids: aspartic acid (Y_Asp), glutamic acid (Y_Glu), asparagine (Y_Asn), serine (Y_Ser), glutamine (Y_Gln), glycine (Y_Gly), histidine (Y_His), arginine (Y_Arg), threonine (Y_Thr), alanine (Y_Ala), proline (Y_Pro), tyrosine (Y_Tyr), valine (Y_Val), methionine (Y_Met), cysteine (Y_Cys), isoleucine (Y_Ile), Leucine (Y_Leu), phenylalanine (Y_Phe), and lysine (Y_Lys). Table 1 and Fig. S2 show 19 yolk free amino acid contents among five genotypes. Significant effects on genotype (P < 0.05) were detected among 8 kinds of free amino acids (Y_Asp, Y_Glu, Y_Ser, Y_Gly, Y_Thr, Y_Tyr, Y_Cys, and Y_Leu). In yolk aspartic acid, NGY (55.7 ± 13.9 µg/mL), which is the highest concentration, was approximately twice as much as the lowest ARA (27.6 ± 7.0 µg/mL). In yolk glycine, NGY, BOR, and ARA were higher than the others. NGY and UKO were the highest and lowest in yolk serine, respectively. On the other hand, yolk cysteine in NGY (1.8 µg/mL) was lower than the other genotypes. NGY and KRH had the lowest and highest content of yolk leucine, respectively. ARA and NGY had greater contents of yolk tyrosine, while UKO was the lowest. KRH and ARA had the highest and lowest content of yolk glutamic acid, respectively. KRH had remarkably higher yolk threonine (71.3 µg/mL) than the other genotypes.

**Albumen free amino acids**

This study detected 19 albumen free amino acids: aspartic acid (A_Asp), glutamic acid (A_Glu), asparagine (A_Asn), serine (A_Ser), glutamine (A_Gln), histidine (A_His), glycine (A_Gly), threonine (A_Thr), arginine (A_Arg), alanine (A_Ala), tyrosine (A_Tyr), valine (A_Val), methionine (A_Met), tryptophan (A_Trp), phenylalanine (A_Phe), isoleucine (A_Ile), leucine (A_Ieu), lysine (A_Lys), and proline (A_Pro). Table 1 and Fig. S3 indicate the results of 19 albumen free amino acid contents among five chicken genotypes. Significant effects of genotype (P < 0.05) were observed on 11 albumen amino acids (A_Asp, A_Glu, A_Asn, A_Ser, A_Gln, A_His, A_Ala, A_Tyr, A_Trp, A_Phe, and A_Ile). Among them, NGY showed the highest values in 7 albumen amino acids (A_Asp, A_Glu, A_Asn, A_Ser, A_Gln, A_Ala, and A_Tyr). In 3 albumen amino acids (A_His, A_Trp, and A_Ile), NGY and UKO were the highest contents. In albumen phenylalanine, UKO (1.53 ± 0.16 µg/mL) was the highest. On the other hand, BOR had the lowest 9 albumen amino acids (A_Asp, A_Glu, A_Asn, A_Ser, A_Gln, A_Ala, A_Tyr, A_Trp, and A_Ile). KRH and ARA had the lowest contents of albumen histidine and phenylalanine, respectively.

**Phenotypic correlation among all egg traits**

Pair-wise correlation coefficients among 48 traits were estimated using all 58 eggs (Fig. 1). In 10 egg traits, there were moderate to strong positive correlations among sizes and weights (EW, LLE, LSE, YW, SW, AW, and ST). In 19 yolk amino acid traits, there were moderate to high positive correlation coefficients among each other, except for yolk cysteine. Among 19 albumen amino acid traits, moderate to strong positive phenotypic correlations were estimated, although there were no correlations between two traits (albumen lysine and albumen proline) and the others. Regardless of positive correlations estimated among traits within each trait category (size and weight traits, yolk amino acid traits, and albumen amino acid traits), there were no or weak phenotypic correlations among the trait categories. Interestingly, there were nearly no correlations between yolk and albumen amino acid traits even in the same metabolites implying that yolk and albumen free amino acids are regulated by partially different genetic basis.

**Fig 1. Phenotypic correlations among all 48 egg traits including yolk and albumen free amino acids.** Ten egg traits, 19 yolk amino acids traits, and 19 albumen amino acids traits from a total of 58 hens (ARA, KRH, UKO, NGY and BOR) were used. Trait abbreviations are shown in Materials and Methods and Results. Pearson’s correlations are expressed by ellipses. Blue and red ellipses indicate positive and negative correlations in each pair, respectively (P < 0.05). Blank cells show not significance.

Phenotypic correlation coefficients among 48 traits were estimated in each genotype (Figs. S4-S8). The similar tendency to phenotypic correlations in all five genotypes (Fig. 1) were basically seen in KRH, UKO, NGY, and BOR (Figs. S5-S8). In ARA (Fig. S4), there were uniquely moderate positive correlations between yolk and albumen amino acid traits.

**Discussion**

In this study, we aimed to investigate the effects of genotype on egg traits, yolk free amino acid traits, and albumen free amino acid traits, using five chicken genotypes (ARA, KRH, UKO, NGY, and BOR). We observed significant effects of genotype on 10 egg traits, 8 yolk amino acids, and 11 albumen amino acids contents. Moderate to strong positive phenotypic correlations among traits within each trait category (size and weight traits, yolk amino acid traits, and albumen amino acid traits), whereas there were nearly no correlations between yolk and albumen amino acid traits even in the same metabolites except for ARA. Thus, these results suggest that many egg traits including yolk and albumen free amino acids can be modified by genetic background under floor rearing environment.

Egg weights in UKO (39.4 ± 2.6 g) and NGY (56.7 ± 4.3 g) in this study were similar to those in UKO (38.5 ± 2.6 g) and NGY (58.3 g) reported by Koketsu and Toyosaki [28] and Nakamura et al. [29]. BOR showed the highest egg weight (61.6 ± 3.6 g) in the five chicken genotypes. However, Kamisoyama et al. [30] and Shimmura et al. [31] reported that egg weights of BOR at 30 weeks of age and 80 weeks of age were 65.1 ± 1.3 g and 66.7 ± 3.1 g, respectively. From these results, egg weight of BOR used in this study will be slightly smaller than the general eggs from Boris Brown. In the Tago Poultry Farm, all the birds were kept in free-range floor rearing environment, which is very far from the conventional cage laying system. Therefore, the environmental difference may affect the phenotypic difference in egg size of Boris Brown.

Of 10 egg traits, the most variety was seen in eggshell redness among five chicken genotypes. Higher eggshell redness values (more than 0) indicate higher degree of redness, while lower the values (less than 0) indicates higher degree of greenness [32]. From the previous studies, brown eggs laid by Rhode Island Red were approximately 11.2 to 14.2 in eggshell redness [22], whereas white eggs laid by White Leghorns were approximately -0.3 to 0.2 [33]. In this study, BOR showed 16.3 ± 1.9 in eggshell redness indicating that the eggshell redness of Boris Brown was quite high. Since ARA showed -2.6 ± 2.2 in eggshell redness, which indicated higher degree of greenness than the other chicken genotypes. On the other hand, the eggshell yellowness of ARA was 15.5 ± 4.2 in this study. Since higher and lower eggshell yellowness values (more and less than 0) indicate higher degree of yellowness and blueness, respectively [32], the present ARA produced yellow eggshell color rather than blue. In general, it is well known that Araucana produces blue eggs [34-36]. Since the ARA egg in this study was close to green rather than blue, the ARA must be a crossbred between purebred Araucana and brown layers. Given that there is some genetic contribution of Brown layer into the present ARA, green colored eggshell will be reasonable.

Yolk and albumen free amino acids among five chicken genotypes were compared to check effects on the genetic differences. This study was conducted under completely the same feeding environment for all hens. Rearing environment was fixed in the free-range floor rearing system, and moreover age at egg sampling was also fixed as 72 weeks of age. Therefore, major parts of the phenotypic variance in this study must be caused by differences of genetic background. This study revealed significant genetic effects in 8 yolk amino acids and 11 albumen amino acids. Of them, the ranking of free amino acid contents was seen as KRH > NGY > ARA > BOR > UKO in yolk and NGY > UKO > ARA, KRH > BOR in albumen. In both yolk and albumen, NGY and BOR showed higher and lower contents, respectively. Our previous study found significant impact on genetic difference in 20 yolk free amino acid traits (Y_Asp, Y_Glu, Y_Asn, Y_Ser, Y_Gln, Y_Gly, Y_His, Y_Arg, Y_Thr, Y_Ala, Y_Pro, Y_GABA, Y_Tyr, Y_Val, Y_Met, Y_Cys, Y_Ile, Y_Leu, Y_Phe, and Y_Lys) and 15 albumen free amino acid traits (A_Asp, A_Glu, A_Ser, A_Gly, A_Thr, A_Arg, A_Ala, A_Tyr, A_Val, A_Met, A_Trp, A_Phe, A_Ile, A_Leu, and A_Pro) using five breeds (Australorp; AUS, NGY, Rhode Island Red; RIR, Shamo; SHA, and UKO) and two F_1_ hybrids (NGYxRIR and SHAxRIR) of chickens under cage rearing system [16]. This study revealed significant effects of genetic difference on 8 yolk free amino acids (Y_Asp, Y_Glu, Y_Ser, Y_Gly, Y_Thr, Y_Tyr, Y_Cys, and Y_Leu) and 11 albumen free amino acids (A_Asp, A_Glu, A_Asn, A_Ser, A_Gln, A_His, A_Ala, A_Tyr, A_Trp, A_Phe, and A_Ile) using five chicken genotypes (ARA, KRH, UKO, NGY, and BOR) under floor rearing system. In comparison with these two experiments, 8 yolk amino acids (Y_Asp, Y_Glu, Y_Ser, Y_Gly, Y_Thr, Y_Tyr, Y_Cys, and Y_Leu) and 8 albumen amino acids (A_Asp, A_Glu, A_Ser, A_Ala, A_Tyr, A_Trp, A_Phe, and A_Ile) were commonly altered by genetic factor suggesting that these free amino acids may have relatively higher heritability estimates than the others.

Essential amino acids for humans are His, Arg, Thr, Tyr, Val, Met, Ile, Leu, Phe, and Lys [37]. Of them, 3 yolk amino acids (Thr, Tyr, and Leu) and 4 albumen amino acids (His, Tyr, Phe, and Ile) were significantly different among five genotypes in this study. KRH showed potential to increase these essential amino acids in yolk, whereas NGY and UKO tended to be higher in albumen. Using these genotypes, essential amino acids enriched eggs may be created. However, this study analyzed free amino acids only. Therefore, the constituent amino acids should be analyzed for accumulating effect of genotype on nutritional values of yolk and albumen. In addition, free amino acids are related to food taste [24, 38]. Zhao et al. [39] and Iwaniak et al. [40] have summarized taste-active amino acids, which are umami taste (Glu, Asp, Ala, and Tyr), sweet taste (Met, Ala, Gly, Pro, Ser, Val, and Lys), sour taste (Asp, Glu, and Lys), bitter taste (Pro, Gly, Val, Leu, Tyr, Phe, His, Lys, Ile, Arg, and Trp), salty taste (Asp), and astringent taste (Lys). This study found significantly altered 6 yolk amino acids (Y_Asp, Y_Glu, Y_Ser, Y_Gly, Y_Tyr, and Y_Leu) and 9 albumen amino acids (A_Asp, A_Glu, A_Ser, A_His, A_Ala, A_Tyr, A_Trp, A_Phe, and A_Ile) indicating that taste added designer eggs will be created by genetic difference in future egg industry. Although amino acid and taste sensor analyses have been conducted with yolk and albumen [23], it is still unclear what degree of difference in free amino acid contents actually affect taste difference in complex food materials such as yolk and albumen. Thus, accumulating knowledge of relationship between taste and amino acids in eggs is needed using human sensory evaluation and instrumental analysis.

It was found that moderate to high phenotypic correlations among free amino acid traits within each yolk and albumen, but no or weak correlations of amino acid traits between yolk and albumen. The relationship of phenotypic correlation is basically supported by Goto et al. [16]. However, this study revealed a unique feature in ARA, which shows moderate positive correlations between yolk and albumen amino acids. This may imply that free amino acid balance in yolk and albumen can be regulated by selecting chicken genotypes. Since egg components have antioxidant properties, antimicrobial activities, immunomodulatory, anticancer, and antihypertensive activities [41, 42], phenotypic correlations among many egg components should be investigated to modify balance in bioactive compounds of eggs in future.

We hypothesized that there are some different features in the free amino acid contents of yolk and albumen between floor and cage rearing systems. Our previous report indicated UKO > NGY in both yolk and albumen amino acid contents under cage rearing system [16]. However, this study revealed NGY > UKO in both yolk and albumen amino acid traits under floor rearing system. Moreover, brown layer (BOR) produced the lowest concentrations of albumen amino acids in this study. Although BOR is frequently used for egg production by floor reared hens in Japan, it may be another choice of genotypes for producing amino acid enriched eggs under floor rearing system.

In summary, genetic factor could affect not only size, weight, and color of the egg but also yolk and albumen free amino acids. Phenotypic correlation analyses revealed unique positive correlations of amino acids between yolk and albumen in ARA. Since there are some possibilities that amino acid performance can be altered by several rearing systems, further investigation of genotype by environment interaction should be conducted.

**Acknowledgements**

This study was supported in part by Grants-in-Aid for the Regional R&D Proposal-Based Program from Northern Advancement Center for Science & Technology of Hokkaido Japan to TG, the Kieikai Research Foundation to TG, and Obihiro City-Obihiro University of Agriculture and Veterinary Medicine Collaboration Project to KN and TG. We are grateful to Drs. Masaaki Hanada and Masafumi Tetsuka for lending laboratory equipment. We thank all members of the Animal Breeding and Genetics Research Group at the Obihiro University of Agriculture and Veterinary Medicine for their continuous supports.

**References**

1. Wang MS, Thakur M, Peng MS, Jiang Y, Frantz LAF, Li M, et al. 863 genomes reveal the origin and domestication of chicken. Cell Res. 2020; 30:693-701. doi: 10.1038/s41422-020-0349-y

2. FAOSTAT. 2020.

http://www.fao.org/poultry-production-products/production/poultry-species/chickens/en/.

3. Tsudzuki M. Japanese native chickens, In: Chang HL, Huang YC (eds). The relationship between indigenous animals and humans in APEC region, The Chinese Society of Animal Science, Tainan, 2003. p. 91–116.

4. Goto T, Shiraishi J, Bungo T, Tsudzuki M. Characteristics of egg-related traits in the Onagadori (Japanese Extremely Long Tail) breed of chickens. J Poult Sci. 2005; 52:81–87. doi: 10.2141/jpsa.0140109

5. Tsudzuki M, Onitsuka S, Akiyama R, Iwamizu M, Goto N, Nishibori M, et al. Identification of quantitative trait loci affecting shank length, body weight and carcass weight from the Japanese cockfighting chicken breed, Oh-Shamo (Japanese Large Game). Cytogenet Genome Res. 2007; 117:288–295. doi: 10.1159/000103190

6. Goto T, Ishikawa A, Nishibori M, Tsudzuki M. A longitudinal quantitative trait loci mapping of chicken growth traits. Mol Genet Genomics. 2019; 294:243–252. doi: 10.1007/s00438-018-1501-y

7. Ono R, Miyachi H, Usui H, Oguchi R, Nishimura K, Takahashi K, et al. Phenotypic analysis of growth and morphological traits in miniature breeds of Japanese indigenous chickens. J Poult Sci. (in press).

8. Bukunmi F, Yusuf H. Analysis of socio-economic factors influencing poultry egg production among poultry farmers in Ondo state, Nigeria. Br J Appl Sci Technol. 2015; 10:1–7. doi: 10.9734/BJAST/2015/12014

9. Hy-Line. 2019. https://www.hyline.com/filesimages/Hy-Line-Products/Hy-Line-Product-PDFs/W-80/80%20COM%20ENG.pdf.

10. Liu Z, Sun C, Yan Y, Li G, Shi F, Wu G, et al. Genetic variations for egg quality of chickens at late laying period revealed by genome-wide association study. Sci Rep. 2018; 8:10832. doi: 10.1038/s41598-018-29162-7

11. Dunn IC, Woolliams JA, Wilson PW, Icken W, Cavero D, Jones AC, et al. Genetic variation and potential for genetic improvement of cuticle deposition on chicken eggs. Genet Sel Evol. 2019. 51:25. doi: 10.1186/s12711-019-0467-5

12. Darwish HYA, Dalirsefat SB, Dong X, Hua G, Chen J, Zhang Y, et al. Genome-wide association study and a post replication analysis revealed a promising genomic region and candidate genes for chicken eggshell blueness. PLoS ONE. 2019; 14:e0209181. doi: 10.1371/journal.pone.0209181

13. Goto T, Tsudzuki M. Genetic mapping of quantitative trait loci for egg production and egg quality traits in chickens: a review. J Poult Sci. 2017; 54:1–12. doi: 10.2141/jpsa.0160121

14. Sirri F, Zampiga M, Soglia F, Meluzzi A, Cavani C, Petracci M. Quality characterization of eggs from Romagnola hens, an Italian local breed. Poult Sci. 2018; 97:4131–4136. doi: 10.3382/ps/pey275

15. Goto T, Mori H, Shiota S, Tomonaga S. Metabolomics approach reveals the effects of breed and feed on the composition of chicken eggs. Metabolites. 2019; 9:224. doi: 10.3390/metabo9100224

16. Goto T, Shimamoto S, Takaya M, Sato S, Takahashi K, Nishimura K, et al. Impact on genetic differences among various chicken breeds on free amino acid contents of egg yolk and albumen. Sci Rep. 2021; 11:2270. doi: 10.1038/s41598-021-81660-3

17. Sadjadi M, Renden JA, Benoff FH, Harper JA. Effects of the blue egg shell allele (*O*) on egg quality and other economic traits in the chicken. Poult Sci. 1983; 62:1717–1720. doi: 10.3382/ps.0621717

18. Edwards HM Jr. Driggers C. Roberta JD, Carmon JL. Studies on the cholesterol content of eggs from various breeds and/or strains of chickens. Poult Sci. 1960; 39: 487–489. doi: 10.3382/ps.0390487

19. Edwards HM Jr. The influence of breed and/or strain on the fatty acid composition of egg lipids. Poult Sci. 1964; 43:751–754. doi: 10.3382/ps.0430751

20. Lordelo M, Cid J, Cordovil CMDS, Alves SP, Bessa RJB, Carolino I. A comparison between the quality of eggs from indigenous chicken breeds and that from commercial layers. Poult Sci. 2020; 99:1768–1776. doi: 10.1016/j.psj.2019.11.023

21. Attia YA, Al-Harthi MA, Korish MA, Shiboo MH. Protein and amino acid content in four brands of commercial table eggs in retail markets in relation to human requirements. Animals. 2020; 10:406. doi: 10.3390/ani10030406

22. Mori H, Takaya M, Nishimura K, Goto T. Breed and feed affect amino acid contents of egg yolk and eggshell color in chickens. Poult Sci. 2020; 99:172–178. doi: 10.3382/ps/pez557

23. Goto T, Shimamoto S, Ohtsuka A, Ijiri D. Analyses of free amino acid and taste sensor traits in egg albumen and yolk revealed potential of value-added eggs in chickens. Anim Sci J. 2021; 92:e13510. doi: 10.1111/asj.13510

24. Kawai M, Sekine-Hayakawa Y, Okiyama A, Ninomiya Y. Gustatory sensation of L-and D-Amino acids in humans. Amino Acids. 2012; 43:2349–2358. doi: 10.1007/s00726-012-1315-x

25. Choudhuri SP, Delay RJ, Delay ER. L-amino acids elicit diverse response patterns in taste sensory cells: a role for multiple receptors. PLoS ONE. 2015; 10:e0130088. doi: 10.1371/journal.pone.0130088

26. Oruna-Concha MJ, Methven L, Blumenthal H, Young C, Mottram DS. Differences in glutamic acid and 5’-ribonucleotide contents between flesh and pulp of tomatoes and the relationship with umami taste. J Agric Food Chem. 2007; 55:5776–5780. doi: 10.1021/jf070791p

27. R Core Team. R: a language and environment for statistical computing. R Foundation for Statistical Computing, Vienna, Austria. 2020. https ://www.R-proje ct.org/.

28. Koketsu M, Toyosaki T. Nutritive constituents of Silky fowl eggs: comparison with hen eggs of White Leghorn origin. Anim Sci J. 2004; 75:67–69. doi: 10.1111/j.1740-0929.2004.00157.x

29. Nakamura K, Ohguchi H, Nagao K, Ida Y, Nakamura A, Ueda J. Elucidation of the processing and taste properties of Nagoya breed eggs. Res Bull Aichi Agric Res Ctr. 2013; 45:105–111.

30. Kamisoyama H, Honda K, Kitaguchi K, Hasegawa S. Transfer of dietary coenzyme Q10 into the egg yolk of laying hens. J Poult Sci. 2010; 47:28–33. doi: 10.2141/jpsa.009037

31. Shimmura T, Hirahara S, Eguchi Y, Uetake K, Tanaka T. Behavior, physiology, performance and physical condition of layers in conventional and large furnished cages in a hot environment. Anim Sci J. 2007; 78:314–322. Doi: 10.1111/j.1740-0929.2007.00441.x

32. Konica Minolta. 2019.

https://www.konicaminolta.com/instruments/knowledge/color/part1/07.html

33. Goto T, Ishikawa A, Goto N, Nishibori M, Umino T, Tsudzuki M. Mapping of main-effect and epistatic quantitative trait loci for internal egg traits in an F_2_ resource population of chickens. J Poult Sci. 2014; 51:375–386. doi: 10.2141/jpsa.0140030

34. Wragg D, Mwacharo JM, Alcalde JA, Wang C, Han JL, Gongora J, et al. Endogenous retrovirus EAV-HP linked to blue egg phenotype in Mapuche fowl. PLoS One. 2013; 8:e71393. doi: 10.1371/journal.pone.0071393

35. Wang XL, Zheng JX, Ning ZH, Qu LJ, Xu GY, Yang N. Laying performance and egg quality of blue-shelled layers as affected by different housing systems. Poult Sci. 2009; 88:1485–1492. doi: 10.3382/ps.2008-00417

36. Noorai RE, Shankar V, Freese NH, Gregorski CM, Chapman SC. Discovery of genomic variations by whole-genome resequencing of the North American Araucana chicken. PLoS One. 2019; 14:e0225834. doi: 10.1371/journal.pone.0225834

37. Wu G. Amino acids: metabolism, functions, and nutrition. Amino Acids. 2009; 37, 1–17. doi: 10.1007/s00726-009-0269-0

38. Kirimura J, Shimizu A, Kimizuka A, Ninomiya T, Katsuya N. Contribution of peptides and amino acids to the taste of foodstuffs. J Agric Food Chem. 1969; 17:689–695. doi: 10.1021/jf60164a031

39. Zhao CJ, Schieber A, Ganzle MG. Formation of taste-active amino acids, amino acid derivatives and peptides in food fermentations – a review. Food Res Int. 2016; 89:39–47. doi: 10.1016/j.foodres.2016.08.042

40. Iwaniak A, Minkiewicz P, Darewicz M, Sieniawski K, Starowicz P. BIOPEP database of sensory peptides and amino acids. Food Res Int. 2016; 85:155–161. doi: 10.1016/j.foodres.2016.04.031

41. Kovacs-Nolan J, Phillips M, Mine Y. Advances in the value of eggs and egg components for human health. J Agric Food Chem. 2005; 53:8421-8431. doi: 10.1021/jf050964f

42. Rehault-Godbert S, Guyot N, Nys Y. The golden egg: nutritional value, bioactivities, and emerging benefits for human health. Nutrients. 2019; 11:684. doi: 10.3390/nu11030684

**Supporting information**

**S1 Figure. Results of Tukey’s HSD test in 10 egg traits using five genotypes.** All combinations among genotypes were tested. Trait abbreviations are expressed in Materials and Methods. P value is shown in each cell. Cells filled by orange meant significant difference in each pair (P < 0.05).

**S2 Figure. Results of Tukey’s HSD test in 19 yolk egg amino acid traits using five genotypes.** All combinations among genotypes were tested. Trait abbreviations are expressed in Results. P value is shown in each cell. Cells filled by orange meant significant difference in each pair (P < 0.05).

**S3 Figure. Results of Tukey’s HSD test in 19 albumen egg amino acid traits using five genotypes.** All combinations among genotypes were tested. Trait abbreviations are expressed in Results. P value is shown in each cell. Cells filled by orange meant significant difference in each pair (P < 0.05).

**S4 Figure. Phenotypic correlations among all 48 egg traits in ARA.** Ten egg traits, 19 yolk amino acids traits, and 19 albumen amino acids traits from 10 hens (ARA) were used. Trait abbreviations are shown in Materials and Methods and Results. Pearson’s correlations are expressed by ellipses. Blue and red ellipses indicate positive and negative correlations in each pair (P < 0.05). Blank cells show not significance.

**S5 Figure. Phenotypic correlations among all 48 egg traits in KRH.** Ten egg traits, 19 yolk amino acids traits, and 19 albumen amino acids traits from 12 hens (KRH) were used. Trait abbreviations are shown in Materials and Methods and Results. Pearson’s correlations are expressed by ellipses. Blue and red ellipses indicate positive and negative correlations in each pair (P < 0.05). Blank cells show not significance.

**S6 Figure. Phenotypic correlations among all 48 egg traits in UKO.** Ten egg traits, 19 yolk amino acids traits, and 19 albumen amino acids traits from 12 hens (UKO) were used. Trait abbreviations are shown in Materials and Methods and Results. Pearson’s correlations are expressed by ellipses. Blue and red ellipses indicate positive and negative correlations in each pair (P < 0.05). Blank cells show not significance.

**S7 Figure. Phenotypic correlations among all 48 egg traits in NGY.** Ten egg traits, 19 yolk amino acids traits, and 19 albumen amino acids traits from 12 hens (NGY) were used. Trait abbreviations are shown in Materials and Methods and Results. Pearson’s correlations are expressed by ellipses. Blue and red ellipses indicate positive and negative correlations in each pair (P < 0.05). Blank cells show not significance.

**S8 Figure. Phenotypic correlations among all 48 egg traits in BOR.** Ten egg traits, 19 yolk amino acids traits, and 19 albumen amino acids traits from 12 hens (BOR) were used. Trait abbreviations are shown in Materials and Methods and Results. Pearson’s correlations are expressed by ellipses. Blue and red ellipses indicate positive and negative correlations in each pair (P < 0.05). Blank cells show not significance.

**Author Contributions**

Conceptualization: Tatsuhiko Goto.

Formal analysis: Kenji Nishimura, Tatsuhiko Goto.

Funding acquisition: Kenji Nishimura, Tatsuhiko Goto.

Investigation: Kenji Nishimura, Daichi Ijiri, Saki Shimamoto, Masahiro Takaya, Akira Ohtsuka, Tatsuhiko Goto.

Project administration: Tatsuhiko Goto.

Supervision: Tatsuhiko Goto.

Writing – original draft: Kenji Nishimura, Tatsuhiko Goto.

Writing – review & editing: Kenji Nishimura, Daichi Ijiri, Saki Shimamoto, Masahiro Takaya, Akira Ohtsuka, Tatsuhiko Goto.
