# Supplementary material for: Genetic effect on free amino acid contents of egg yolk and albumen using five different chicken genotypes under floor rearing system
Source: PLoS One. 2021 Oct 8;16(10):e0258506. doi: 10.1371/journal.pone.0258506 (PMC8500412; doi:10.1371/journal.pone.0258506)
Supplement: Attachment — Submitted filename: Response to Reviewers 2 - Nishimura et al.docx [file pone.0258506.s010.docx]

PONE-D-21-14031R1
Genetic effect on free amino acid contents of egg yolk and albumen using five different chicken genotypes under floor rearing system
PLOS ONE

Dear Dr. Tatsuhiko Goto,

Thank you for submitting your manuscript to PLOS ONE. After careful consideration, we feel that it has merit but does not fully meet PLOS ONE’s publication criteria as it currently stands. Therefore, we invite you to submit a revised version of the manuscript that addresses the points raised during the review process.

Please submit your revised manuscript by September 15 2021 11:59PM. If you will need more time than this to complete your revisions, please reply to this message or contact the journal office at [plosone@plos.org](mailto:plosone@plos.org). When you're ready to submit your revision, log on to <https://www.editorialmanager.com/pone/> and select the 'Submissions Needing Revision' folder to locate your manuscript file.

Please include the following items when submitting your revised manuscript:

- A rebuttal letter that responds to each point raised by the academic editor and reviewer(s). You should upload this letter as a separate file labeled 'Response to Reviewers'.
- A marked-up copy of your manuscript that highlights changes made to the original version. You should upload this as a separate file labeled 'Revised Manuscript with Track Changes'.
- An unmarked version of your revised paper without tracked changes. You should upload this as a separate file labeled 'Manuscript'.

If you would like to make changes to your financial disclosure, please include your updated statement in your cover letter. Guidelines for resubmitting your figure files are available below the reviewer comments at the end of this letter.

If applicable, we recommend that you deposit your laboratory protocols in protocols.io to enhance the reproducibility of your results. Protocols.io assigns your protocol its own identifier (DOI) so that it can be cited independently in the future. For instructions see: <http://journals.plos.org/plosone/s/submission-guidelines#loc-laboratory-protocols>. Additionally, PLOS ONE offers an option for publishing peer-reviewed Lab Protocol articles, which describe protocols hosted on protocols.io. Read more information on sharing protocols at <https://plos.org/protocols?utm_medium=editorial-email&utm_source=authorletters&utm_campaign=protocols>.

We look forward to receiving your revised manuscript.

Kind regards,

Ewa Tomaszewska, DVM Ph.D
Academic Editor
PLOS ONE

Journal Requirements:

Please review your reference list to ensure that it is complete and correct. If you have cited papers that have been retracted, please include the rationale for doing so in the manuscript text, or remove these references and replace them with relevant current references. Any changes to the reference list should be mentioned in the rebuttal letter that accompanies your revised manuscript. If you need to cite a retracted article, indicate the article’s retracted status in the References list and also include a citation and full reference for the retraction notice.

[Note: HTML markup is below. Please do not edit.]

Reviewers' comments:

Reviewer's Responses to Questions

**Comments to the Author**

1. If the authors have adequately addressed your comments raised in a previous round of review and you feel that this manuscript is now acceptable for publication, you may indicate that here to bypass the “Comments to the Author” section, enter your conflict of interest statement in the “Confidential to Editor” section, and submit your "Accept" recommendation.

Reviewer #1: All comments have been addressed

Reviewer #2: (No Response)

2. Is the manuscript technically sound, and do the data support the conclusions?

The manuscript must describe a technically sound piece of scientific research with data that supports the conclusions. Experiments must have been conducted rigorously, with appropriate controls, replication, and sample sizes. The conclusions must be drawn appropriately based on the data presented.

Reviewer #1: Yes

Reviewer #2: Partly

3. Has the statistical analysis been performed appropriately and rigorously?

Reviewer #1: Yes

Reviewer #2: Yes

4. Have the authors made all data underlying the findings in their manuscript fully available?

The [PLOS Data policy](http://www.plosone.org/static/policies.action#sharing) requires authors to make all data underlying the findings described in their manuscript fully available without restriction, with rare exception (please refer to the Data Availability Statement in the manuscript PDF file). The data should be provided as part of the manuscript or its supporting information, or deposited to a public repository. For example, in addition to summary statistics, the data points behind means, medians and variance measures should be available. If there are restrictions on publicly sharing data—e.g. participant privacy or use of data from a third party—those must be specified.

Reviewer #1: (No Response)

Reviewer #2: Yes

5. Is the manuscript presented in an intelligible fashion and written in standard English?

PLOS ONE does not copyedit accepted manuscripts, so the language in submitted articles must be clear, correct, and unambiguous. Any typographical or grammatical errors should be corrected at revision, so please note any specific errors here.

Reviewer #1: Yes

Reviewer #2: No

6. Review Comments to the Author

Please use the space provided to explain your answers to the questions above. You may also include additional comments for the author, including concerns about dual publication, research ethics, or publication ethics. (Please upload your review as an attachment if it exceeds 20,000 characters)

Reviewer #1: The authors made the suggested revisions to the manuscript. In addition, in situations of disagreement, they have provided comprehensive arguments that allow us to accept the manuscript in its present form

Reviewer #2: Thank you very much for revising your manuscript. The manuscript requires following points to be addressed in another round of revision:

1. The authors have made many changes according to the specific comments. However, I was unable to see any those changes highlighted or in different font color. This made the evaluation of revisions in revised manuscript difficult to follow. Any changes made should have been highlighted or changed font color even if suggested or incorporated by the reviewer.
[Authors’ response] Editorial Manager required both clean version and revised manuscript with track changes (changed font color by red). So, we have submitted the two versions of first revised manuscripts. However, we have not expressed all the things which you indicated comprehensive grammatical suggestions with track changes. Therefore, we indicated all the first round revised points by red letters and the second round points by blue letters. If you evaluate all the things throughout the manuscript, we are very happy.

2. The language is consistently poor. From the authors' responses, it seems that they did not consult any native English speaking colleague or a language editing service. The authors should consider to improve the language to make it for better understanding of readers.
[Authors’ response] We had our native English-speaking colleague help with revising our manuscript before submitting. Since there were so many grammar errors, we revised the manuscript entirely (blue letters). In addition, we could receive your comprehensive concrete advices using WORD track change function in the first round of revise. Your concrete advices make our manuscript better for potential readers. We really appreciate it.

3. The authors have been unable to convince why there was not a comparison with a representative laying hen breed as a control group. Also, most of the breeds they evaluated were hybrids (e.g., Araucana, Kurohisui, and Boris Brown). The authors exert that Boris Brown (a hybrid cross) is better for making a comparison. Even if the authors want to compare other breeds with BOR, it should be declared somewhere in the manuscript.
[Authors’ response] Thank you very much. We described “Since BOR (GHEN Corporation, Japan) is the world’s brown egg layer (Hy-Line Brown; Hy-Line International, USA), we selected BOR as representative hen for the comparison.” (L 102-104).

4. The authors have been unable to remove the discussion part related to floor rearing system. The authors should understand that floor rearing system was not used as a factor. Since the floor rearing system was used across the groups (breeds in this case), the discussion related to floor rearing system becomes irrelevant and should be deleted.

[Authors’ response] Thank you very much. We will analyze and report the effect of rearing systems on egg amino acids in the future. According to your suggestions, we deleted a whole paragraph in discussion from this paper.

The deleted paragraph is shown below.

~~We hypothesized that there are some different features in the free amino acid contents of yolk and albumen between floor and cage rearing systems. Our previous report indicated UKO > NGY in both yolk and albumen amino acid contents under cage rearing system [17]. However, this study revealed NGY > UKO in both yolk and albumen amino acid traits under floor rearing system. Moreover, Brown layer (BOR) produced the lowest concentrations of albumen amino acids in this study. Although BOR is frequently used for egg production by floor reared hens in Japan, it may be another choice of genotypes for producing amino acid enriched eggs under floor rearing system.~~

7. PLOS authors have the option to publish the peer review history of their article ([what does this mean?](https://journals.plos.org/plosone/s/editorial-and-peer-review-process#loc-peer-review-history)). If published, this will include your full peer review and any attached files.


If you choose “no”, your identity will remain anonymous but your review may still be made public.


**Do you want your identity to be public for this peer review?** For information about this choice, including consent withdrawal, please see our [Privacy Policy](https://www.plos.org/privacy-policy).

Reviewer #1: No

Reviewer #2: No

[NOTE: If reviewer comments were submitted as an attachment file, they will be attached to this email and accessible via the submission site. Please log into your account, locate the manuscript record, and check for the action link "View Attachments". If this link does not appear, there are no attachment files.]

While revising your submission, please upload your figure files to the Preflight Analysis and Conversion Engine (PACE) digital diagnostic tool, <https://pacev2.apexcovantage.com/>. PACE helps ensure that figures meet PLOS requirements. To use PACE, you must first register as a user. Registration is free. Then, login and navigate to the UPLOAD tab, where you will find detailed instructions on how to use the tool. If you encounter any issues or have any questions when using PACE, please email PLOS at [figures@plos.org](mailto:figures@plos.org). Please note that Supporting Information files do not need this step.

*In compliance with data protection regulations, you may request that we remove your personal registration details at any time.* [*(Remove my information/details)*](https://www.editorialmanager.com/pone/login.asp?a=r)*. Please contact the publication office if you have any questions.*
